# Hepatitis E virus infection in 6-month-old pigs in Taiwan

**DOI:** 10.1038/s41598-020-74034-8

**Published:** 2020-10-09

**Authors:** Ming-Hui Liao, Fang-Tzy Wu, Huimin Bai, Yen Hai Doan, Jyh-Yuan Yang, Naokazu Takeda, Masamichi Muramatsu, Tian-Cheng Li

**Affiliations:** 1 Department of Veterinary Medicine, College of Veterinary Medicine, National Pingtung University of Science and Technology, No. 1, Hseuh-Fu Road, Nei Pu, Pingtung, 91201 Taiwan; 2 Department of Post-Baccalaureate Veterinary Medicine, College of Medical and Health Science, Asia University, No. 500, Liou-Feng Road, Wufeng Dist., Taichung City, 41354 Taiwan; 3 Center for Research, Diagnostics and Vaccine Development, Taiwan Centers for Disease Control, No.161, Kun-Yang Street, Taipei City, 11561 Taiwan; 4 Department of Basic Medicine and Forensic Medicine, Baotou Medical College, Jianshe Road 31, Baotou, 014060 Inner Mongolia People’s Republic of China; 5 Research Institute for Microbial Diseases, Osaka University, Suita, Osaka 565-0781 Japan; 6 Department of Virology II, National Institute of Infectious Diseases, 4-7-1 Gakuen, Musashi-murayama, Tokyo, 208-0011 Japan; 7 Department of Environmental Parasitology, Tokyo Medical and Dental University, M&D Tower 16F, 1-5-45 Yushima, Bunkyo-ku, Tokyo, 113-8519 Japan

**Keywords:** Genetics, Zoology

## Abstract

Hepatitis E virus (HEV) is the causative agent of acute hepatitis E. Genotype 3 (G3) and 4 (G4) HEV have recently been identified in and isolated from swine as the main HEV genotypes worldwide. However, there is limited information on HEV infection status among pigs in Taiwan, especially pigs in the stage before transportation to the slaughterhouse. To determine the frequency of HEV infection among pigs in Taiwan, we detected and quantified HEV RNA contained in 295 fecal specimens collected from 6-month-old pigs bred in 30 pig farms located in 8 counties. We found that 25.1% (74/295) of the fecal specimens were positive for HEV RNA by a quantitative real-time reverse transcription-polymerase chain reaction, and the copy number ranged from 2.3 × 10^3^ to 2.08 × 10^7^ copies/g. Amplification of a 338 bp sequence in ORF2 was achieved in 16 of 74 HEV RNA-positive samples, and their nucleotide sequences were determined. Two HEV sequences appeared to belong to subtype 3a of G3 and the remaining 14 HEV sequences belonged to subtype 4b of G4 (G4b). The entire genome sequence of two G4b HEVs was obtained by next-generation sequence analyses, and the phylogenetic analyses indicated that unique G4b HEVs were circulating in pig farms in Taiwan. In the present study, we found that both G3 and G4 HEVs were circulating in Taiwanese pig farms and G4b was the predominant subtype. In addition, the relatively high detection frequency of HEV RNA in the 6-month-old pigs indicated that Taiwanese pigs just before transportation to the slaughterhouse are at risk of carrying HEVs, and thus thorough cooking or heating of pork meat or organs is needed before consumption in Taiwan and possibly in other countries as well.

## Introduction

Hepatitis E virus (HEV) is the cause of self-limiting acute or fulminant type E hepatitis, and is primarily transmitted by an oral-fecal route^1,2^. Hepatitis E is a public health concern not only in many Asian and African countries where sanitation conditions are insufficient but also in industrialized countries. Recently, increasing incidence of hepatitis E associated with zoonotic infection has drawn public attention in industrialized countries^3^.

Recent studies have demonstrated that HEV is a quasi-enveloped virus^4^ with a positive-sense single-stranded RNA genome. It belongs to the family *Hepeviridae*, which includes two genera, *Orthohepevirus* and *Piscihepevirus,* based on the nucleotide sequence divergence^5^. The *Orthohepevirus* is further subdivided into four distinct species, *Orthohepevirus A–D*^5^. The species *Orthohepevirus A* is grouped into 8 genotypes, G1 to G8, mainly according to the animal from which HEV is isolated—namely, humans, monkeys, swine, wild boar, deer, camels, mongooses or rabbits.

Five genotypes of HEV, G1, G2, G3, G4, and G7, belonging to *Orthohepevirus A* are known to infect humans^5,6^, with G1 and G2 infecting humans exclusively, while G3, G4, and G7 HEVs infect both humans and animals^7,8^. The relatively high mortality rate among G1 HEV-infected pregnant women (5–25%) is a latent threat in endemic regions, and is a unique feature of HEV infection^9,10^. G3 and G4 HEV are distributed worldwide, infecting humans, swine, wild boar and rabbits and are responsible for sporadic and zoonotic infections^3,11^.

Swine are thought to be the main reservoir of G3 and G4 HEV^12^. Because HEV-infected pigs excrete large quantities of HEV into the feces, zoonotic transmission of HEV could occur through direct contact with pigs. In fact, the antibody positive rate against HEV was found to be 1.51 times higher in veterinarians handling pigs than in normal blood donors^13^, and was also higher among swine farmers than the general population^14^. Because HEV replicates in the liver and the transient viremia is associated with the dissemination of HEV into muscle and other tissues, consumption of uncooked or undercooked liver, meat or related products from HEV-infected pigs might confer a risk of HEV transmission in humans^15^. Therefore, we investigated the current infection status of HEV in the pigs just before transportation to the slaughterhouse. Our findings should be useful for the risk assessment and management of viral hepatitis due to HEV.

## Materials and methods

### Sample collection

**Figure 1:**
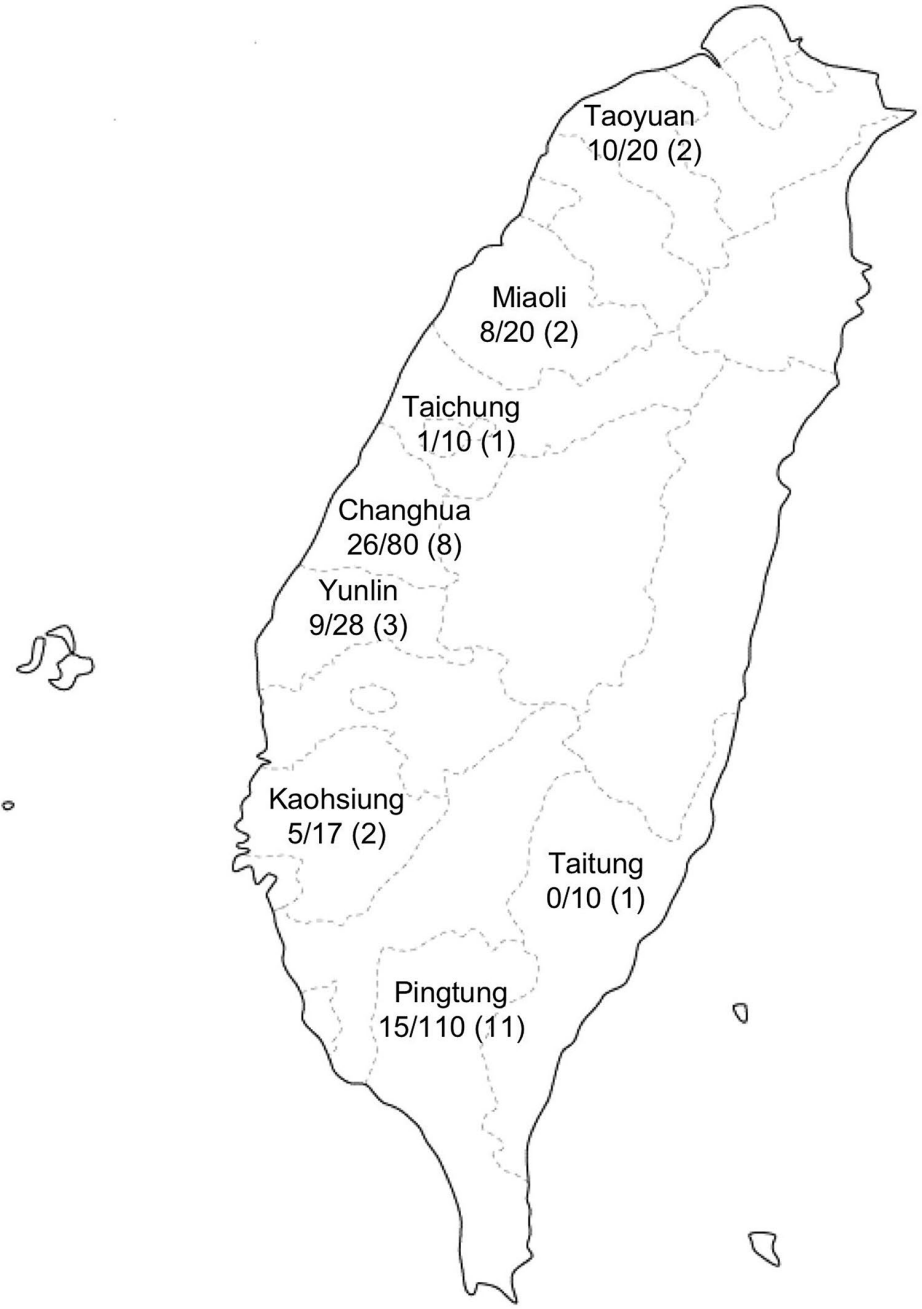
Geographical distribution of sampling counties in Taiwan. The swine fecal specimens collected in each county are shown as “HEV RNA positive numbers/collected samples numbers (farm numbers)”. A free map was downloaded from https://www.freemap.jp/itemFreeDlPage.php?b=asia&s=taiwan, and modified by using Adobe Photoshop CS2.

A total of 295 swine fecal specimens were collected from 30 commercial farms (F1 to F30) in Taiwan from January 12 to December 13, 2015 (Table 1). The swine farms were located in 8 counties: Pingtung (F1-4, F6, F9-11, F14, F17, and F29), Changhua (F13, F15, F16, F18, F20, F27, F28, and F30), Miaoli (F25, F26), Yunlin (F12, F23, and F24), Taoyuan (F21 and F22), Taitung (F19), Taichung (F5), and Kaohsiung (F7 and F8). Ten samples were collected from each farm, except 2 farms where 7 (F8) and 8 (F23) samples were collected (Fig. 1 and Table 1). All of the pigs were 6 months old and therefore in the terminal fattening stage before shipping. Three grams of fecal specimens were directly collected from individual swine and diluted with 10 mM phosphate-buffered saline (PBS) to prepare a 10% (w/v) suspension. The suspension was shaken at 4 °C for 1 h, clarified by centrifugation at 10,000 × g for 30 min, passed through a 0.45 µm membrane filter (Millipore, Bedford, MA), and stored at − 80 °C until use^16^. The experiments were reviewed and approved by the Taiwan Centers for Disease Control (CDC) ethics committee and all of the animal experiments were carried out according to the *Guides for Animal Experiments Performed at Taiwan CDC*.

**Table 1:** Detection of HEV RNA in swine fecal specimens.

Farm	Collection date	Collection area	Positive/Total (%)	Genotype
F1	Jan 12, 2015	Pingtung	0/10 (0)*	
F2	Jan 29, 2015	Pingtung	1/10 (10)	
F3	Feb 2, 2015	Pingtung	7/10 (70)	4b (2)**
F4	Feb 3, 2015	Pingtung	1/10 (10)	
F5	Feb 6, 2015	Taichung	1/10 (10)	
F6	March 2, 2015	Pingtung	0/10 (0)	
F7	March 9, 2015	Kaohsiung	3/10 (30)	4b (1)
F8	March 11, 2015	Kaohsiung	2/7 (42.8)	4b (1)
F9	Apr 9, 2015	Pingtung	0/10 (0)	
F10	Apr 13, 2015	Pingtung	0/10 (0)	
F11	May 4, 2015	Pingtung	1/10 (10)	
F12	May 7, 2015	Yunlin	0/10 (0)	
F13	May 20, 2015	Changhua	1/10 (10)	
F14	June 1, 2015	Pingtung	1/10 (10)	
F15	June 17, 2015	Changhua	9/10 (90)	4b (3)
F16	June 20, 2015	Changhua	1/10 (10)	
F17	July 2, 2015	Pingtung	1/10 (10)	
F18	July 21, 2015	Changhua	0/10 (0)	
F19	Aug 13, 2015	Taitung	0/10 (0)	
F20	Aug 21, 2015	Changhua	1/10 (10)	
F21	Sep 10, 2015	Taoyuan	3/10 (30)	
F22	Oct 2, 2015	Taoyuan	7/10 (70)	4b (6)
F23	Oct 6, 2015	Yunlin	4/8 (50)	4b (1)
F24	Oct 15, 2015	Yunlin	5/10 (50)	
F25	Nov 2, 2015	Miaoli	4/10 (40)	3a (2)
F26	Nov 6, 2015	Miaoli	4/10 (40)	
F27	Nov 13, 2015	Changhua	4/10 (40)	
F28	Dec 3, 2015	Changhua	6/10 (60)	
F29	Dec 7, 2015	Pingtung	3/10 (30)	
F30	Dec 11, 2015	Changhua	4/10 (40)	
Total			74/295 (25.1)	

*HEV RNA-positive rates detected by real-time RT-qPCR.

**Number of samples used to determine the nucleotide sequence.

### Quantitative real-time reverse transcription-polymerase chain reaction (real-time RT-qPCR) for the detection of HEV

HEV RNA was extracted from 200 µl of the 10% suspension using a MagNA Pure LC Total Nucleic Acid Isolation Kit (Roche Applied Science, Mannheim, Germany) and eluted with 50 µl RNase free water according to the manufacturer’s recommendations. To determine the copy numbers of HEV RNA, a TaqMan assay was performed with a 7500 FAST Real-Time PCR System (Applied Biosystems, Foster City, CA) using TaqMan Fast Virus 1-step Master Mix (Applied Biosystems). Real-time RT-qPCR targeting a 70 bp region of ORF3/ORF2 was carried out with a forward primer (5′-GGTGGTTTCTGGGGTGAC-3′), a reverse primer (5′- AGGGGTTGGTTGGATGAA-3′), and a probe (5′-FAM-TGATTCTCAGCCCTTCGC-TAMRA-3′) under the following conditions: 5 min incubation at 50 °C, 20 s incubation at 95 °C, and 40 cycles of 3 s at 95 °C and 30 s at 60°C^17^. A tenfold serial dilution of the full-length G3 HEV RNA (10^7^ to 10^1^ copies) was used as the standard to quantitate the copy numbers. G3 HEV was originally isolated from the fecal specimen of a pig and cultured in a human hepatocarcinoma cell line, PLC/PRF/5. cDNA was produced from isolated virus RNA, and the full genome (AB740232) was cloned into pUC19 under the T7 promoter^18^. The capped G3 HEV RNA was synthesized using an mMESSAGE mMACHINE T7 kit (Ambion, Austin, TX) and the copy number was calculated based on the RNA concentration and molecular weight. Amplification data were collected and analyzed with Sequence Detector software ver. 1.3 (Applied Biosystems). The detection limit was 10^3^ copies/ml.

### RT-PCR for amplification of the HEV genome

Reverse transcription was performed with a high-capacity cDNA reverse transcription kit (ABI Applied Biosystems, Foster City, CA) at 25 °C for 10 min, 37 °C for 120 min and 85 °C for 5 min in a 20 µl reaction mixture containing 1 µl of reverse transcriptase, 2 µl of the random primer, 1 µl of RNase inhibitor, 2 µl of 10 × RT buffer, 0.8 µl of 10 mM deoxynucleoside triphosphates, 8 µl of RNA, and 5.2 µl of distilled water^19^.

A nested polymerase chain reaction (PCR) was performed to amplify a portion of the open reading frame 2 (ORF2) genome. The first PCR was carried out with an external forward primer, HEV-F1 (5′-TAYCGHAAYCAAGGHTGGCG-3′), and an external reverse primer, HEV-R2 (5′-TGYTGGTTRTCRTARTCCTG-3′). The amplification was carried out for 35 cycles (95 °C for 30 s, 55 °C for 45 s, and 72 °C for 90 s) after a denaturation at 95 °C for 60 s and followed by a final extension at 72 °C for 7 min. Two microliters of the first PCR product were used for the nested PCR with an internal forward primer, HEV-F2 (5′-GGBGTBGCNGAGGAGGAGGC-3′), and an internal reverse primer, HEV-R1 (5′-CGACGAAATYAATTCTGTCG-3′), under the same amplification conditions as used for the first PCR. The monkey fecal samples collected before- and post-G1 HEV infection were used as the negative and positive control for RT-PCR, respectively. The detection limit was determined to be 10^4^ copies/ml by real-time RT-qPCR. The nested PCR products with 378 bp nucleotides were separated by electrophoresis on 2% agarose gels^20^.

### HEV genome sequencing

The PCR products were purified using a QIAquick PCR purification kit (Qiagen, Hilden, Germany), and the nucleotide sequencing was carried out with primers HEV-F2 and HEV-R1 using an ABI 3130 Genetic Analyzer Automated Sequencer (Applied Biosystems, Foster City, CA) and a BigDye Terminator Cycle Sequencing Ready Reaction kit (Applied Biosystems) according to the manufacturer’s instructions. Sequence analysis was performed using the Genetyx ver.11.0.4 software program (Genetyx Corp., Tokyo).

### Next-generation sequence analysis (NGS)

The entire genome sequences were determined by NGS as described previously^21^. Briefly, the viral RNA was extracted from the 10% fecal specimens, and a 200 bp fragment library was constructed with a NEBNext Ultra RNA Library Prep Kit for Illumina version 2.0 (New England Biolabs, Ipswich, MA) according to the manufacturer’s instructions. Library purification was done using Agencourt AMPure XP magnetic beads (Beckman Coulter, Brea, CA). A 151-cycle paired-end read sequencing run was carried out on a MiSeq desktop sequencer (Illumina, San Diego, CA) using an MiSeq Reagent Kit version 2 (300 cycles). Sequence data were analyzed using CLC Genomics Workbench Software version 7.5.1 (CLC Bio, Aarhus, Denmark).

### Phylogenetic analyses

Phylogenetic trees with 1,000 bootstrap replicates were generated by the neighbor-joining method based on the partial ORF2 sequence (338 bp) or entire HEV genome. Bootstrap values of 95 or higher were considered statistically significant for the grouping^22^. The nucleotide sequence alignment was performed using Clustal X 1.81. The genetic distance was calculated by Kimura's two-parameter model^23^.

## Results

### Characterization of HEV in pig fecal specimens

To determine the frequency of HEV infection among pig populations in Taiwanese farms, we detected and quantified HEV RNA in 295 fecal specimens collected from 6-month-old pigs bred in 30 pig farms in 8 counties in Taiwan (Fig. 1). HEV RNA was detected in 23 out of 30 farms: 0% (0/10) in Taitung, 10% (1/10) in Taichung, 13.6% (15/110) in Pingtung, 29.4% (5/17) in Kaohsiung, 32.1% (9/28) in Yunlin, 32.5% (26/80) in Changhua, 40% (8/20) in Miaoli, and 50% (10/20) in Taoyuan County (Table 1). We found that 25.1% (74 of 295) of the fecal specimens were positive for HEV RNA by real-time RT-qPCR, and the copy number ranged from 2.3 × 10^3^ to 2.1 × 10^7^ copies/g.

**Figure 2:**
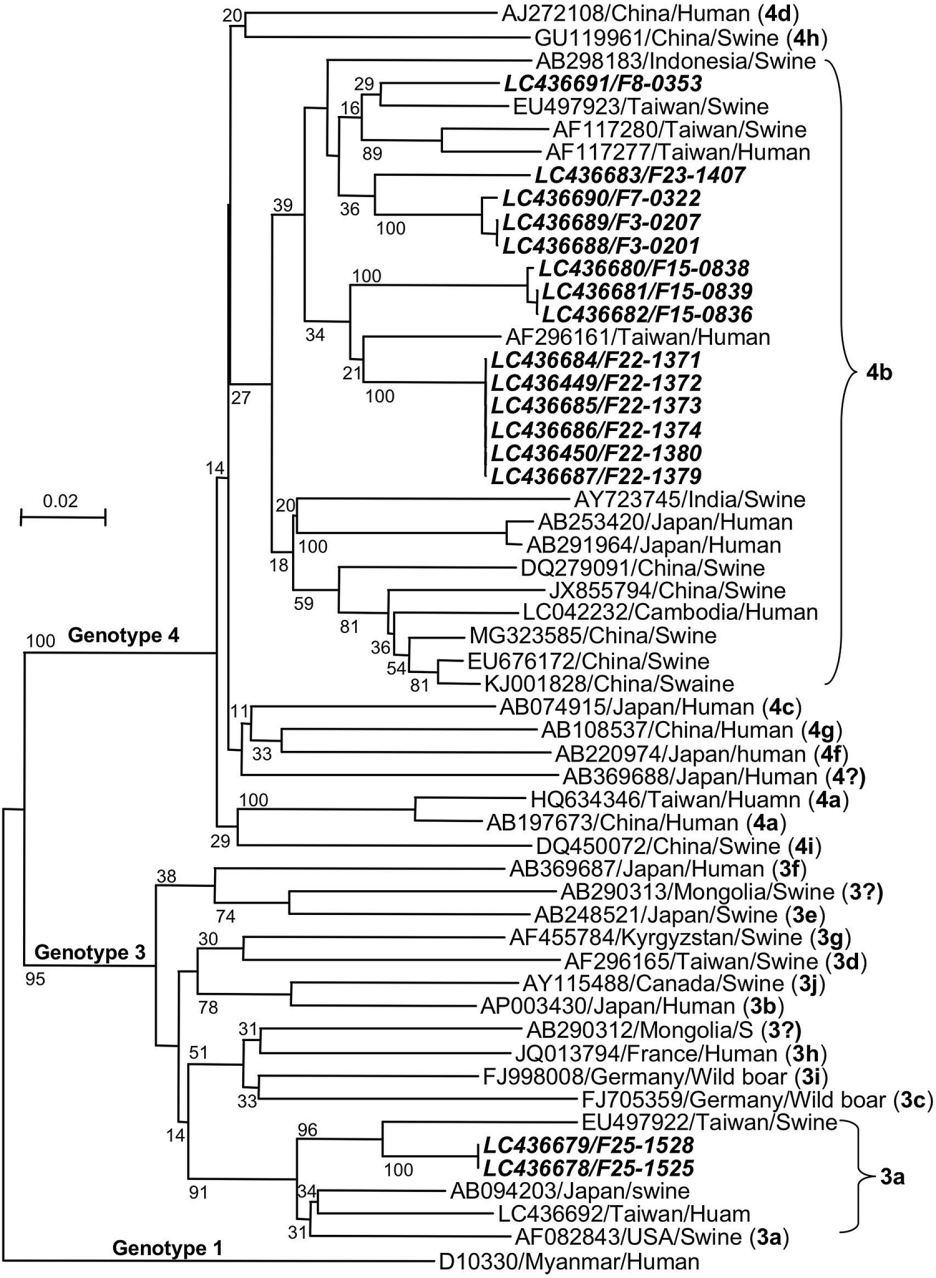
Phylogenetic analyses based on the partial ORF2 sequences. A phylogenetic tree with 1,000 bootstrap replicates was generated based on the partial HEV ORF2 sequence (338 bp). The scale bar indicates the nucleotide substitutions per site. The numbers on the branches represent the bootstrap values. The reference sequences were labeled as “GenBank accession no./country/animal”. HEVs detected in the present study are labeled as “GenBank accession no./farm no.-pig no.” and shown in bold italic letters.

Amplification of a 378 bp sequence in ORF2 was achieved in 16 of 74 HEV RNA-positive samples, and their nucleotide sequences were determined (GenBank accession nos. LC436678-LC436692, LC436449, and LC436450). Phylogenetic analyses indicated that 14 sequences belonged to G4, subtype 4b (G4b) (Fig. 2); these were 2 sequences from farm F3 in Pingtung; 1 from farm F7 and 1 from farm F8 in Kaohsiung; 3 from farm F15 in Changhua; 6 from farm F22 in Taoyuan; and 1 from farm F23 in Yunlin County (Fig. 1, Table 1). The inter-farm nucleotide sequence identities between the 6 farms were 89.5% to 99.7%, while the intra-farm nucleotide sequence identities were as high as 99.4% to 100%. These 14 G4b HEVs shared 90.4% to 94.0% nucleotide sequence identity with those detected in the serum from hepatitis patients (AF296161 and AF117277) and 90.7% to 93.5% nucleotide sequence identity with those detected in the serum from swine (EU497922 and AF117280) in Taiwan, respectively. The remaining 2 HEV sequences collected from farm F25 in Miaoli County were identical and belonged to G3, subtype 3a (G3a), sharing 90.5% to 92.9% nucleotide sequence identity with the G3a HEV sequences detected in Taiwan, Japan and the USA (Fig. 2). These results demonstrated that several genetically different HEVs were circulating in the pig farms in Taiwan.

### The complete genome of G4b HEV

All 16 samples that were positive for HEV RNA by RT-PCR and real time RT-qPCR were further analyzed by NGS, and the entire genome sequences were obtained from 2 of the fecal specimens, F22-1372 and F22-1380. Both HEV RNAs consisted of 7230 nucleotides (nt), and a poly (A) tail and the 5′- and 3′-terminal untranslated regions containing 26 and 70 nucleotides (GenBank accession nos. LC436449 and LC436450). Both HEV RNAs encoded 3 open reading frames (ORFs), ORF1 (nt 27-5141, 1,704 aa), ORF2 (nt 5180-7162, 660 aa), and ORF3 (nt 5166-5510, 114 aa). We found 4 nucleotide differences between them (C1091T, C4355T, C6355T and T6715C), and the nucleotide sequence identity was 99.9%, although the amino acid sequences of ORF1, ORF2 and ORF3 were identical.

**Figure 3:**
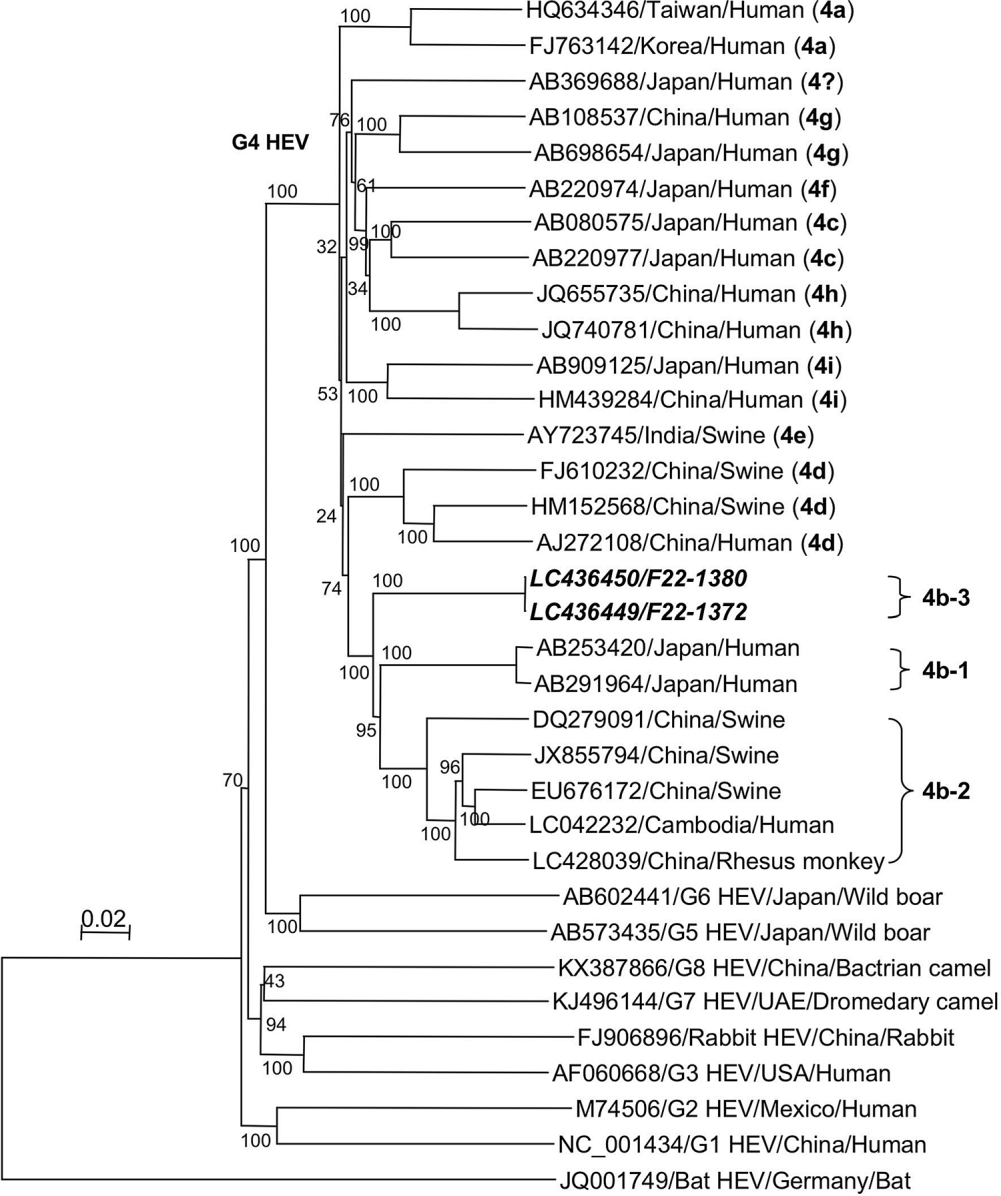
Phylogenetic analyses based on the entire genome. A phylogenetic tree was generated based on the entire genome of HEVs. This figure is labeled as in Fig. 2.

Phylogenetic analyses based on the entire genome demonstrated that these 2 HEVs, F22-1372 and F22-1380, belonged to G4b (Fig. 3). When we compared these 2 Taiwanese HEVs with 7 known G4b strains isolated in Japan, China and Cambodia, they were further separated into 3 clusters: G4b-1, which included 2 strains isolated from patients in Japan; G4b-2, which included 4 strains detected in pigs and rhesus monkeys in China and human patients in Cambodia; and G4b-3, which included 2 Taiwanese HEVs. The 2 Taiwanese HEVs analyzed in the present study shared 86.9–86.9% and 87.5–87.9% nucleotide sequence identities with G4b-1 and G4b-2, respectively, and formed a separate cluster, suggesting that G4b HEV is genetically diverse, and unique G4b HEVs were circulating in pig farms in Taiwan.

## Discussion

Swine is a major reservoir of G3 and G4 HEV, and consumption of pig-derived foods is a potential source of zoonotic HEV infection^24−28^. Generally, HEV infection occurs after the weaning stage, and HEV RNA is detected mainly in serum samples in 3- to 4-month-old pigs in the farms^29,30^. The anti-HEV IgG-positive rates were shown to be as high as 90%, and no HEV RNA was detected in the serum samples in 6-month-old pigs^30^. However, Yazaki et al. tested packages of raw pig liver sold in grocery stores as food in Hokkaido, Japan, and found that 7 of 363 (1.9%) packages were positive for HEV RNA^31^. In the United Kingdom, the prevalence of the antibodies to HEV was 92.8% in pigs at the time of slaughter, and HEV RNA was detected in 15% of cecal contents and 3% of plasma samples in these pigs^32^. Moreover, the entire genome of G3 HEV was detected in the liver of a fattening pig in Switzerland^33^. In addition, the HEV RNA genome was detected in pork products such as meats, liver sausages and liver paté in Switzerland, Canada and France^26,34,35^. These results suggested that slaughter pigs and pork products are at risk of carrying HEV to humans. Further studies to explore the status of HEV infection may help to elucidate the potential risk of type E hepatitis deriving from the pigs before transportation to the slaughterhouse.

Because the rearing period of pigs is 6 months, we collected the fecal specimens from 6-month-old pigs in 30 farms in Taiwan, and found that 23 out of 30 farms were exposed to HEV and 25.1% of the pigs were positive for HEV RNA. This unexpectedly high prevalence of HEV RNA in the 6-month-old pigs obtained in the present study confirmed that the pigs before transportation to the slaughterhouse have a high risk for the spread of HEV infection. Although we exclusively examined HEV RNA by using fecal specimens, other tissues, such as meats, intestine or liver, must also be examined for HEV RNA after transportation to the slaughterhouse in order to evaluate the contamination of HEV.

Although a total of 74 fecal samples were positive for HEV RNA by real-time RT-qPCR, the amount of HEV RNA was lower than 10^4^ copies/g in most of the samples. These results indicated that the copy numbers of the HEV genomes in the feces of 6-month-old pigs were low. However, we detected copy numbers as high as over 10^7^ copies/g of RNA in two pigs (F22-1372 and F22-1380) in Taoyuan County, suggesting the possibility of super spreaders even in the final fattening stage of the pigs. In addition, the entire HEV genome was obtained from the feces of those two pigs. Phylogenetic trees were constructed based on both the partial ORF2 sequence (338 bp) and the entire HEV genome, and they showed that F22-1372 and F22-1380 were segregated into the subtype G4b. Therefore, there is no discrepancy in their constellation between the trees.

G3 and G4 HEV have been detected in hepatitis patients and pigs in Taiwan^36−39^, but the genetic information was limited, particularly for the entire genome of HEV. Our phylogenetic analyses based on the partial ORF2 sequences of the 16 HEVs revealed that both G3 and G4 HEV were circulating in pig farms in Taiwan. The G3a genome was detected in only 1 pig farm, while G4b was detected in 6 farms, suggesting that G4 HEV is more prevalent than G3 HEV in the Taiwanese pig farms.

In summary, our findings demonstrated the high prevalence of HEV in 6-month-old pigs in Taiwan, and suggested that pigs before transportation to the slaughterhouse are at a high risk of carrying HEV to humans. Since HEV could be inactivated by heating^40,41^, thorough cooking or heating is highly recommended before consumption of pork, pork liver, pork intestine or other related products to reduce the zoonotic infection due to HEV.
